# VITAMIN D AND ASTHMA: A RELATIONSHIP TO BE CLARIFIED

**DOI:** 10.1590/1984-0462/;2018;36;3;00020

**Published:** 2018

**Authors:** Gustavo Falbo Wandalsen, Dirceu Solé

**Affiliations:** a Escola Paulista de Medicina, Universidade Federal de São Paulo, São Paulo, SP, Brasil.

The most well-known role of vitamin D in the human body is played in bone metabolism and mineralization.^1^ In recent years, however, an increasing interest in the role of vitamin D in the pathophysiology and management of asthma has emerged.

Experimental studies conducted with humans and animals have reported that vitamin D acts in a variety of ways in the immune and respiratory systems. In innate immunity, vitamin D may increase and modulate inflammatory response against bacteria, viruses and fungi.^2^ In adaptive immunity, it impacts the modulation of antigen presentation by dendritic cells. Vitamin D also plays a role in regulating the response of regulatory T lymphocytes (Tregs), which are known to be relevant in asthma.^2^

Vitamin D has also been shown to reduce bronchial smooth muscle hypertrophy and goblet cell hyperplasia, as well as subepithelial collagen deposition and fibroblast activity, typical of the airway remodeling observed in asthma.^2^

Numerous population studies have reported association between asthma and vitamin D. Compared with non-asthmatics, children and adults with asthma have higher incidence of vitamin D insufficiency.^3^ Among asthmatics, serum 25-hydroxyvitamin D-25 levels-25(OH)D-are negatively correlated with the severity of the disease, leading to greater need for corticosteroids, with degree of airway remodeling and bronchial hyperresponsiveness, as well as serum levels of immunoglobulin E (IgE).^2^ ^,^ ^3^

Despite strong evidence that vitamin D deficiency can adversely affect asthma, the opposite is also possible. Reduced exposure to sun associated with limited physical activities, chronic inflammation (of the airways) and comorbidities such as obesity also have a negative influence on vitamin D levels. Thus, the association between asthma and vitamin D is multifactorial and determined by mutual feedback mechanisms.^2^

Genetic evidence also associates asthma with vitamin D. Several genes linked to asthma may be regulated by vitamin D, and polymorphisms in the vitamin D receptors are associated with increased risk of developing the disease.^2^ The present issue of *Revista Paulista de Pediatria* brings to you an interesting article by Santos et al.^4^ that addressed some aspects of the potential relationship between vitamin D and asthma. Vitamin D levels and vitamin D receptor polymorphisms have been assessed in two groups of asthmatics (with and without inhaled corticosteroid treatment) and compared with a control group. The main finding of the study was the description of a new polymorphism (CDX2) in the vitamin D gene, which is associated with asthma in children.^4^ In a recent meta-analysis, other polymorphisms were associated with asthma in children, but the findings vary depending on the ethnicity studied,^5^ which reinforces the need for additional local studies on the subject.

Two vitamin D deficiency situations have been traditionally identified: deficiency, defined as serum levels of 25(OH)D below 20 ng/mL and insufficiency, characterized by 25(OH)D levels between 20 and 30 ng/mL^2^. In the study by Santos et al., 98% of the children evaluated had inadequate vitamin D levels according to these criteria^4^. The current debates on the most appropriate reference levels of 25(OH)D are very important to aid in clinical interpretation of these findings.

Mounting evidence of the role of vitamin D in the pathophysiology of asthma and the possibility of improving asthma control by simply and inexpensively supplementing it inspired the investigation of the impacts of vitamin D supplementation in the prevention of exacerbations and control of asthma in both children and adults. Several clinical trials have already been performed, and systematic reviews with meta-analysis have evaluated their clinical effects in children.^6^ ^,^ ^7^ Despite the heterogeneity and quality of studies, reduction in exacerbation rates but not in other clinical outcomes have been observed in a review,^6^ as well as a decrease in rates of exacerbations requiring systemic corticosteroid use in another paper.^7^

Some studies indicate that vitamin D may play a role in the management of asthma in adults. Vitamin D deficiency is cited as one of the mechanisms responsible for corticosteroid resistance in severe asthmatics. In a proof-of-concept study, severe adult asthmatics with proven resistance to oral steroids had a better response to oral prednisolone after four weeks of vitamin D supplementation.^8^ In adults with non-allergic asthma, vitamin D supplementation was able to reduce inflammation of airways in cases of severe eosinophilic inflammation.^9^

These evidences, however, are not enough to support indiscriminate supplementation of vitamin D in asthmatics, so further studies and better evidence on the subject are needed.^10^ The relationship between asthma and vitamin D was proven quite complex and presents many factors still to be clarified.
